# Continuous Femoral Nerve Block Versus Continuous Epidural Analgesia for Postoperative Analgesia in Knee Surgeries: A Randomized Controlled Trial

**DOI:** 10.7759/cureus.94548

**Published:** 2025-10-14

**Authors:** Siddardan Pasupathy, Chandraleela Sundararajan, Radhakrishnan A, H. Riaz Fathima, Manikandan A

**Affiliations:** 1 Anaesthesiology, Sri Venkateshwaraa Medical College Hospital & Research Centre, Puducherry, IND; 2 Orthopaedics, Sri Venkateshwaraa Medical College Hospital & Research Centre, Puducherry, IND

**Keywords:** continuous epidural analgesia, continuous femoral nerve block, knee surgery, postoperative analgesia, randomized controlled trial

## Abstract

Background: Postoperative pain in knee surgeries hampers rehabilitation and increases morbidity. Continuous femoral nerve block (CFNB) and continuous epidural analgesia (CEA) are widely used, but their comparative efficacy remains uncertain.

Objective: This study aimed to compare the analgesic efficacy, hemodynamic stability, and rescue analgesic requirements of CFNB and CEA in patients undergoing knee surgeries.

Methods: In this double-blind randomized controlled trial, 70 American Society of Anesthesiologists (ASA) grades I and II patients (18-60 years) undergoing elective knee surgeries were randomized into two groups. Group A received CFNB with 0.2% ropivacaine at 6 ml/hr. Group B received CEA with 0.2% ropivacaine at 6 ml/hr. Visual Analogue Scale (VAS) pain scores were recorded at 0-48 hours postoperatively. Hemodynamic parameters and rescue analgesia were also assessed.

Results: Both groups showed comparable VAS scores throughout 48 hours (p > 0.05). At six hours, VAS was 4.06 ± 1.19 in Group A vs. 4.29 ± 0.67 in Group B (p = 0.32). Hemodynamic parameters remained stable, and rescue analgesic requirements were not significantly different.

Conclusion: CFNB and CEA provide equivalent postoperative analgesia and hemodynamic stability in knee surgeries. Either technique may be chosen based on patient profile and institutional preference.

## Introduction

Knee arthroplasties, including total knee arthroplasty (TKA), anterior cruciate ligament (ACL) reconstruction, and other orthopedic arthroplasties, have been well-established procedures to regain functional and pain-free status and improve the quality of life in patients with degenerative or traumatic knee disease [1]. Pain relief following such surgeries is crucial postoperatively, and inadequate analgesia leads to delayed rehabilitation, longer hospital stays, and higher morbidity. Multimodal analgesia methods have been used conventionally, with a combination of systemic analgesics, regional blocks, and local anesthetics being given to achieve optimal pain relief [2].

Regional anesthetic methods like continuous femoral nerve block (CFNB) and continuous epidural analgesia (CEA) have become extremely popular all over the world because they have been shown to deliver better analgesia, lower opioid intake, and allow for early mobilization. The use of CFNB compared to CEA, though, is a debated topic since each of the methods has multiple pros and cons based on the clinical setup and patient [3,4].

The burden of knee osteoarthritis and traumatic knee injuries in India is substantial, reflecting global trends. With a rapidly aging population and increasing obesity rates, the prevalence of knee osteoarthritis is on the rise, particularly among individuals over the age of 50 [5]. In India, studies estimate that nearly 28% of adults over 50 suffer from symptomatic knee osteoarthritis [6]. TKA procedures are also witnessing significant growth, with projections indicating a steady increase in demand. While exact national statistics are limited, estimates suggest that over 200,000 TKA procedures are performed annually in India, a figure expected to rise due to demographic shifts and lifestyle changes [7]. The increasing burden of osteoarthritis, combined with a high incidence of road traffic accidents, one of the leading causes of traumatic knee injuries, has further contributed to the rising need for surgical interventions [8]. These trends align with global patterns, as seen in the United States and other parts of Asia, emphasizing the growing orthopedic healthcare demand in India.

Optimal pain management is critical in knee arthroplasty, and 30%-60% of patients receiving TKA experience severe postoperative pain [9]. Poor control of pain is not only an obstacle to postoperative physical therapy and functional status but also a risk for developing chronic pain in up to 20% of patients. Ideal analgesic protocols should thus be centered on achieving maximum surgical success and patient satisfaction [10].

This study aimed to compare CFNB and CEA in a well-defined population of patients undergoing knee surgery with uniform protocols and assessments. By comparison, not just of pain scores but also of functional recovery, rate of complications, and patient satisfaction, this study aimed to provide clinicians with solid evidence on which to base analgesic choice in the perioperative period.

## Materials and methods

Study design

The study was a prospective double-blind randomized controlled study conducted at Sri Venkateshwaraa Medical College Hospital and Research Centre, Puducherry, India, from May 2023 to February 2025.

Sample size

The sample size was calculated with a confidence interval of 95%, a power of 80%, the ratio between the groups was 1, and a mean difference of 1.6 from the study by Brindha et al. [11]. Sample size was calculated using OpenEpi software, version 3 (Dean et al., 2013; https://www.openepi.com/). A total of 70 patients (35 in each group) were selected for the study.

Study population

The study population included 70 consenting American Society of Anesthesiologists (ASA) grade I and II patients from 18 to 60 years of age undergoing elective knee surgeries with the following inclusion and exclusion criteria.

Inclusion criteria

Patients who were ASA grade I and II patients of either sex between the ages of 18 and 60 years, undergoing elective knee surgeries, were included in the study.

Exclusion criteria

Exclusion criteria included refusal to participate; allergy to local anesthetics; morbid obesity; polytrauma; infection at the injection site; spinal deformities; and a history of femoral bypass surgery.

Randomization

The patients were divided into two groups (Group A and Group B) by a computer-generated randomization technique. A sealed envelope for group allocation was prepared by a person who was not involved in performing the procedure, data collection, and analysis. The envelopes were opened and viewed only by the anesthesiologist performing the procedure (who was not involved in data collection).

Blinding (double blinding)

The observer (PI), who was blinded to group allocation, recorded all the necessary data for the study, and the patients recruited were unaware of group allocation. To maintain blinding and minimize bias, identical dressings were applied to both the epidural and femoral regions in both groups, regardless of the actual site of the catheter placement.

Study groups

Group A received CFNB with 0.2% ropivacaine at 6 ml/hr. Group B received CEA with 0.2% ropivacaine at 6 ml/hr. Both groups had 35 participants each.

Materials

The materials used in the study included an infusion pump; an ultrasound machine (SonoSite Edge 2, FUJIFILM SonoSite Inc., Bothell, WA, USA) with a high-frequency linear probe; ultrasound jelly; sterile film; a probe cover; a sterile spinal tray; a sterile block tray; a 26G Quincke needle; an 18G Tuohy needle; and an epidural catheter kit.

Standard monitors were used, including electrocardiogram (ECG), pulse oximetry, and non-invasive blood pressure (NIBP) monitoring.

Methodology

After getting approval from the Institutional Human Ethical Committee of the institution (reference no: 52/SVMCH/IEC-Cert/May 23), the study was registered at the Clinical Trials Registry-India (CTRI/2023/11/059809). Written informed consent of participating patients was obtained. Patients were equally divided into two groups (Group A and Group B) of 35 each by a computer-generated randomization technique. The allocation sequence was concealed from the investigator by the Serially Numbered Opaque Sealed Envelope (SNOSE) method. The investigator and patient were both blinded to the group allocations. This was given to a senior anesthesiologist who performed the block technique at the end of the surgery. Pre-anesthetic check-ups and routine investigations were done preoperatively. Patients satisfying the inclusion criteria were selected and counselled about the risks and benefits involved in the study. After obtaining written informed consent, patients were enrolled in the study. Nil per oral status was confirmed, the procedure was explained, and the patient was informed to communicate about the perception of any pain or discomfort during the surgery, which was recorded using a visual analogue scale. Patients were premedicated with tablet alprazolam 0.25 mg, tablet pantoprazole 40 mg orally, and tablet metoclopramide 10 mg on the night before surgery. Patients were transferred to the operating room approximately 30 minutes before the scheduled procedure. All emergency resuscitation equipment, anesthetic drugs, and monitors were prepared in advance. Arrangements for both regional and general anesthesia, along with the ultrasound machine for nerve block guidance, were made and verified for readiness prior to induction. The patients were divided into two groups (Group A and Group B) by a computer-generated randomization technique. A sealed envelope for group allocation was prepared by a person who is not involved in performing the procedure, data collection, and analysis. Baseline vitals such as pulse rate, non-invasive blood pressure, saturation in room air, respiratory rate (RR), and ECG pattern were recorded. Intravenous access was secured using an 18-G IV cannula, and a crystalloid infusion of lactated Ringer’s solution or normal saline (RL/NS) was started at 100 mL/hr.

In Group A (CFNB), patients were positioned supine with the operative limb slightly abducted (10°-20°) and minimally externally rotated so the lateral foot rested on the table. Under strict aseptic precautions, after draping the femoral region, an 18-G Tuohy needle was introduced under ultrasound guidance using a SonoSite Edge 2 machine with a high-frequency linear probe to identify the femoral nerve. The needle was advanced toward the femoral nerve, and the catheter was carefully threaded into the femoral nerve sheath. A small saline bolus was injected to confirm proper catheter placement, after which the catheter was secured firmly in position.

In Group B (CEA) for epidural analgesia, the patient was seated or made to lie on their side in a lateral position. The epidural space was confirmed using loss of resistance (LOR) or the hanging drop technique, following which an epidural catheter was inserted to the required depth; 3 ml of 1.5% lignocaine with adrenaline (1:200000) was given as a test dose, which can rule out both subarachnoid and intravascular injection.

In all the patients in both the groups, after femoral or epidural catheter insertion, subarachnoid block was given in a sitting or lateral position in the L3‑L4 intervertebral space using a 25G Quincke Babcock spinal needle with 0.5% hyperbaric bupivacaine 3 ml (15 mg) as a standard volume across both the groups and also to achieve a dense sensory block to a level of T10. The level of sensory block following spinal anesthesia was assessed using the pinprick and cold swab technique in the cephalad direction to determine the extent of sensory loss. To maintain blinding and minimize bias, identical dressings were applied to both the epidural and femoral regions in both groups, regardless of the actual site of catheter placement. An infusion was started at 6 ml/h postoperatively when the Visual Analog Scale (VAS) was ≥ 4. The dose was titrated to keep VAS <4 (with a minimum rate of 2 ml/h and a maximum rate of 10 ml/h). If VAS ≥ 4 occurred despite the maximum rate of infusion, a rescue analgesic was given.

Postoperative pain was assessed using a VAS (VAS score: 0 = no pain, 1 to 4 = mild pain, 4 to 7 = moderate pain, 8 to 10 = severe pain, and 10 = worst possible pain). A continuous infusion of ropivacaine 0.2% was started at 6 ml/hour when VAS was ≥4 (maximum infusion rate was 10 ml/h and minimum infusion rate was 2 ml/h). The infusion was continued for 48 hours after surgery. Patients' vital parameters, like pulse rate, blood pressure, SpO_2_, and RR, were assessed intraoperatively and postoperatively for 48 hrs. The patient's pain assessment was done by the VAS scale. Pain assessment was done at 0, one, and two hours and every fourth hour for 48 hours. Patients were also monitored for any adverse effects.

In cases where patients experienced breakthrough pain despite the primary analgesic technique, rescue analgesia was administered to ensure adequate pain control. In this study, intravenous paracetamol injection at a dose of 15 mg/kg was used as rescue analgesia. These agents were selected for their efficacy and safety profiles in the postoperative setting, aiming to provide prompt pain relief while minimizing adverse effects.

Statistical analysis

Data entry was done by using Microsoft Excel (Microsoft Corporation, Redmond, WA), and data analysis was done by IBM SPSS Statistics software for Windows, version 23.0 (IBM Corp., Armonk, NY). Description of continuous variables was expressed using mean and standard deviation, and categorical variables in frequency and percentage. For continuous variables such as age, weight, blood pressure, heart rate, VAS scores, and analgesic requirements, independent two-sample t-tests were used to compare groups. 

Qualitative data

Tests were used to assess the difference between the two proportions and the association between the study variables, like gender and ASA physical status. The chi-square test was employed. All the tests were two-tailed, and a p-value < 0.05 is shown to have a significant relationship.

## Results

The mean age of Group A was 33.60 ± 8.82 years, while Group B had a mean age of 35.63 ± 9.47 years (p = 0.3567), indicating no statistically significant difference. The p-value is based on Student’s t-test.

The height of patients in Group A (158.66 ± 6.12 cm) was comparable to that of Group B (159.49 ± 5.98 cm, p = 0.5680). The weight and BMI were also statistically similar, with Group A having a mean weight of 66.14 ± 5.38 kg and a BMI of 26.34 ± 2.48 kg/m², compared to Group B with 64.97 ± 6.14 kg and 25.59 ± 2.59 kg/m², respectively (p = 0.3995 for weight, 0.2202 for BMI). The p-value is based on Student’s t-test. These values indicate that both groups had similar baseline anthropometric characteristics.

In terms of gender distribution, Group A had 18 males and 17 females, while Group B had 23 males and 12 females (p = 0.3318). The p-value is based on the chi-square test.

**Figure 1:**
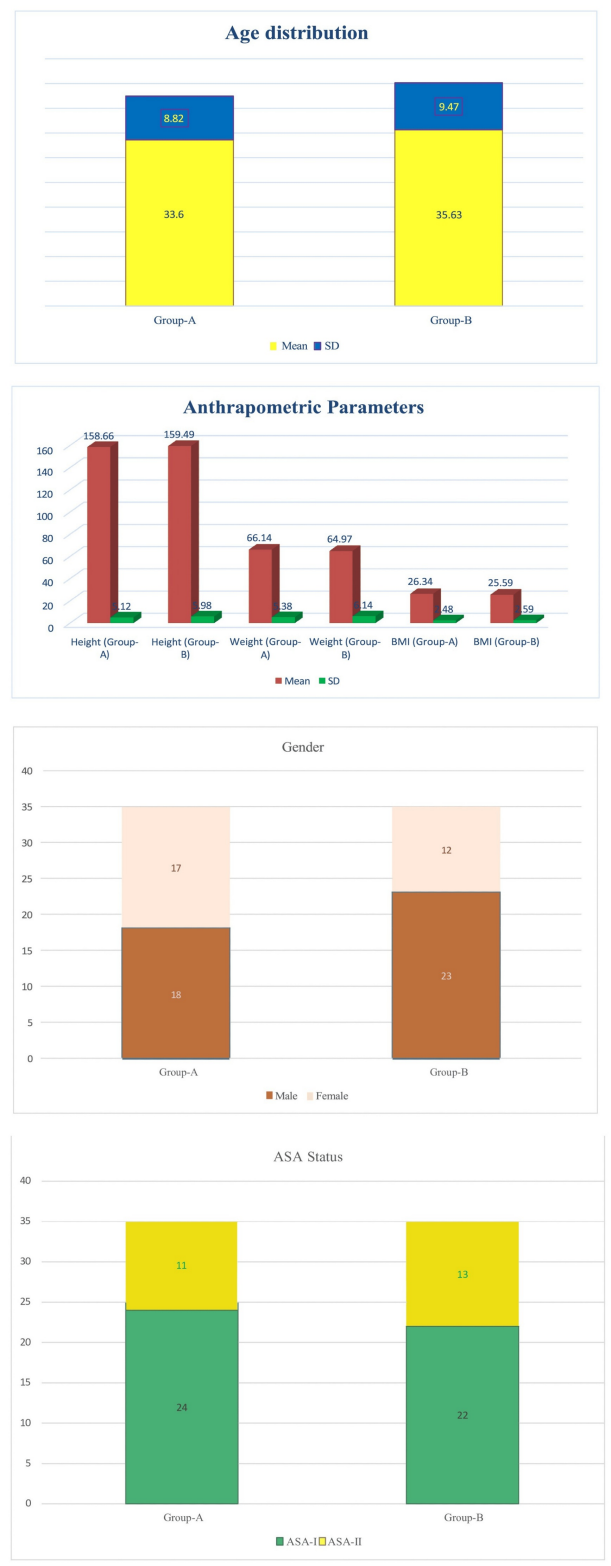
Demographic parameters: age, anthropometry, gender, BMI, and ASA grade The p-value is based on Student’s t-test for age, anthropometry, and BMI. The p-value is based on the chi-square test for gender and ASA grade. ASA: American Society of Anesthesiologists

The proportion of ASA I and ASA II patients was also similar between the groups, with ASA I: 24 vs. 22 patients and ASA II: 11 vs. 13 patients (p = 0.6480). The p-value is based on the chi-square test (Figure 1 & Table 1).

**Table 1:** Demographic parameters: age, gender, BMI, and ASA grade The p-value is based on Student’s t-test for age, anthropometry, and BMI. The p-value is based on the chi-square test for gender and ASA grade. ASA: American Society of Anesthesiologists

Parameter		Group A		Group B		p-value
		Mean	SD (+/-)	Mean	SD (+/-)	
Age (years)		33.60	8.82	35.63	9.47	0.36
Height (cm)		158.66	6.12	159.49	5.98	0.57
Weight (kg)		66.14	5.38	64.97	6.14	0.40
BMI (kg/m^2^)		26.34	2.48	25.59	2.59	0.22
Gender	Male	18		23		0.33
	Female	17		12		
ASA	I	24		22		0.80
	II	11		13		

**Figure 2:**
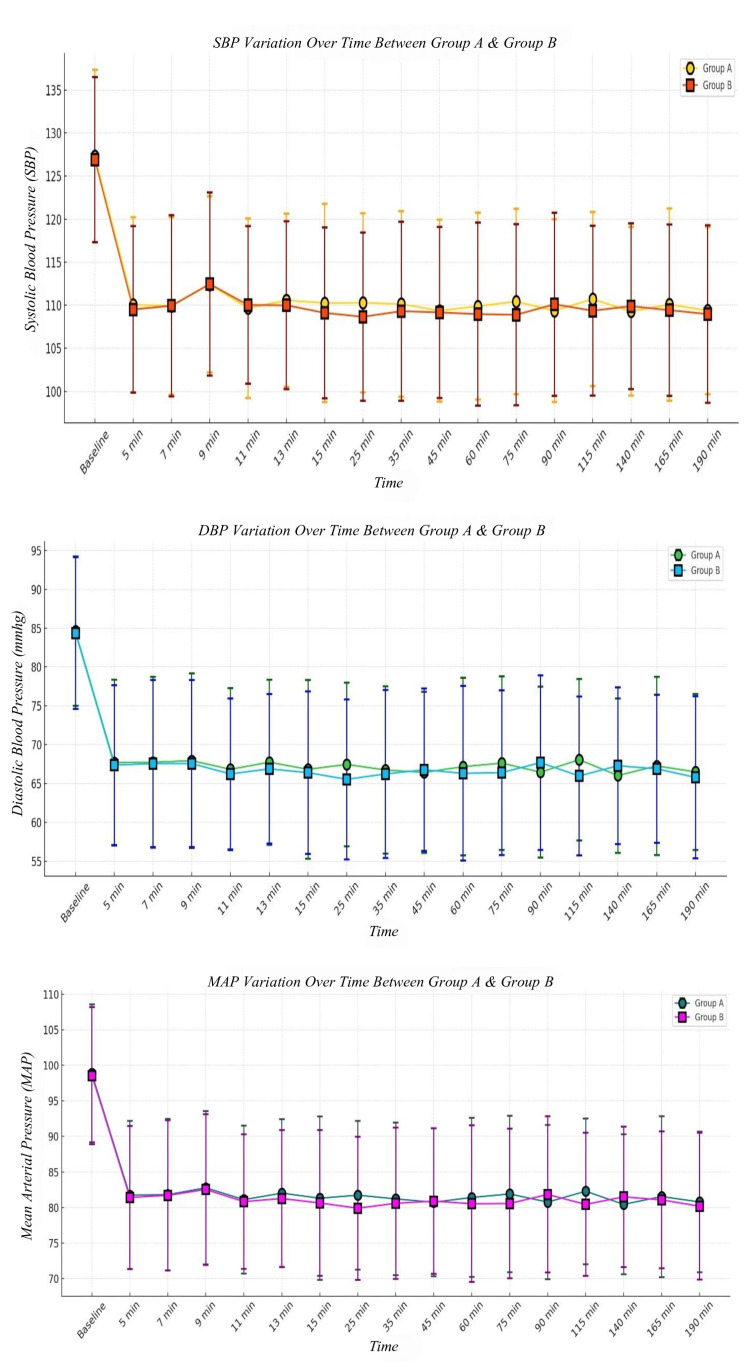
Intraoperative systolic, diastolic, mean arterial blood pressures The p-value is based on Student’s t-test. SBP: systolic blood pressure; DBP: diastolic blood pressure; MAP: mean arterial pressure; min: minutes

The baseline systolic blood pressure (SBP) was 127.31 ± 10.02 mmHg in Group A and 126.91 ± 9.59 mmHg in Group B (p = 0.8650), showing no significant difference. Throughout the intraoperative period, SBP remained comparable between the groups, with values fluctuating between 109 and 112 mmHg in both groups at different time points. The highest variation was seen at nine minutes (112.43 ± 10.24 mmHg in Group A vs. 112.46 ± 10.63 mmHg in Group B, p = 0.9904), yet the difference remained non-significant across all measurements. These findings indicate that intraoperative SBP was similar between the two groups. The p-value is based on Student’s t-test (Table 2 & Figure 2).

**Table 2:** Intraoperative SBP (mmHg) The p-value is based on Student’s t-test. SBP: systolic blood pressure; min: minutes

SBP	Group A		Group B		p-value
	Mean	SD (+/-)	Mean	SD (+/-)	
Baseline	127.31	10.02	126.91	9.59	0.86
5 min	110.06	10.17	109.51	9.65	0.82
7 min	109.91	10.29	109.94	10.51	0.99
9 min	112.43	10.24	112.46	10.63	0.99
11 min	109.66	10.43	110.03	9.13	0.87
13 min	110.57	10.07	110.00	9.74	0.81
15 min	110.26	11.49	109.11	9.93	0.65
25 min	110.29	10.40	108.66	9.77	0.50
35 min	110.14	10.78	109.31	10.39	0.74
45 min	109.37	10.57	109.17	9.93	0.93
60 min	109.89	10.84	108.97	10.65	0.72
75 min	110.43	10.75	108.89	10.52	0.57
90 min	109.37	10.59	110.11	10.62	0.77
115 min	110.71	10.10	109.37	9.86	0.57
140 min	109.29	9.78	109.89	9.62	0.80
165 min	110.09	11.18	109.43	9.94	0.79
190 min	109.38	9.72	108.97	10.29	0.86

The baseline diastolic blood pressure (DBP) was 84.63 ± 9.60 mmHg in Group A and 84.37 ± 9.76 mmHg in Group B (p = 0.9109), showing negligible differences. DBP values during surgery remained within a narrow range of 66-68 mmHg, with no statistically significant differences between the groups (p-values > 0.05 for all time points). The p-value is based on Student’s t-test (Table 3 & Figure 2).

**Table 3:** Intraoperative DBP (mmHg) The p-value is based on Student’s t-test. DBP: diastolic blood pressure; min: minutes

DBP	Group A		Group B		p-value
	Mean	SD (+/-)	Mean	SD (+/-)	
Baseline	84.63	9.60	84.37	9.76	0.91
5 min	67.69	10.68	67.37	10.30	0.89
7 min	67.74	10.99	67.57	10.74	0.95
9 min	67.94	11.25	67.57	10.74	0.88
11 min	66.83	10.44	66.23	9.72	0.80
13 min	67.74	10.63	66.89	9.64	0.73
15 min	66.83	11.51	66.40	10.45	0.87
25 min	67.46	10.53	65.54	10.30	0.44
35 min	66.74	10.76	66.23	10.83	0.84
45 min	66.43	10.37	66.77	10.45	0.89
60 min	67.17	11.42	66.31	11.25	0.75
75 min	67.63	11.18	66.40	10.59	0.64
90 min	66.46	11.02	67.69	11.23	0.65
115 min	68.06	10.38	65.97	10.23	0.39
140 min	66.03	9.94	67.29	10.08	0.60
165 min	67.26	11.48	66.89	9.54	0.88
190 min	66.50	10.03	65.79	10.43	0.77

Mean arterial pressure (MAP) at baseline was 98.86 ± 9.70 mmHg in Group A and 98.55 ± 9.66 mmHg in Group B (p = 0.8938). Intraoperatively, MAP fluctuated between 80 and 83 mmHg in both groups, with the lowest recorded MAP at 11 minutes (81.10 ± 10.40 mmHg in Group A vs. 80.83 ± 9.48 mmHg in Group B, p = 0.9100). Since all p-values were greater than 0.05, there was no significant difference in intraoperative MAP control between the two groups. The p-value is based on Student’s t-test (Table 4 & Figure 2).

**Table 4:** Intraoperative MAP (mmHg) The p-value is based on Student’s t-test. MAP: mean arterial pressure; min: minutes

MAP	Group A		Group B		p-value
	Mean	SD (+/-)	Mean	SD (+/-)	
Baseline	98.86	9.70	98.55	9.66	0.89
5 min	81.75	10.45	81.42	10.04	0.89
7 min	81.80	10.68	81.70	10.58	0.97
9 min	82.77	10.77	82.53	10.61	0.92
11 min	81.10	10.40	80.83	9.48	0.91
13 min	82.02	10.41	81.26	9.63	0.75
15 min	81.30	11.47	80.64	10.25	0.80
25 min	81.73	10.44	79.91	10.08	0.46
35 min	81.21	10.72	80.59	10.64	0.81
45 min	80.74	10.39	80.90	10.24	0.95
60 min	81.41	11.19	80.53	11.02	0.74
75 min	81.90	11.00	80.56	10.52	0.60
90 min	80.76	10.84	81.83	11.00	0.68
115 min	82.28	10.25	80.44	10.07	0.45
140 min	80.45	9.83	81.49	9.89	0.66
165 min	81.53	11.33	81.07	9.63	0.85
190 min	80.79	9.88	80.19	10.33	0.80

**Figure 3:**
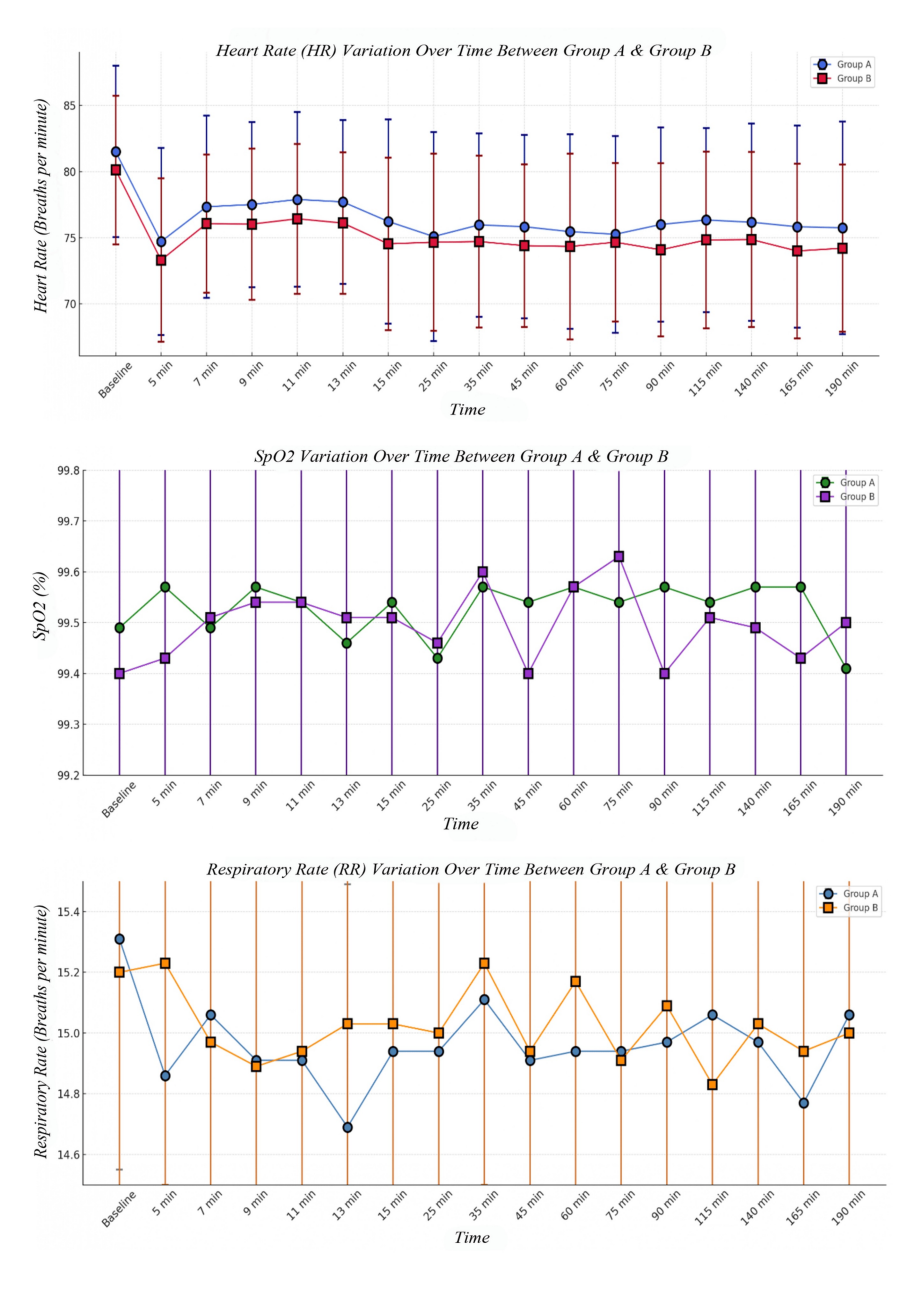
Intraoperative heart rate, Spo2, and respiratory rate The p-value is based on Student’s t-test. min: minutes

Baseline heart rate (HR) was 81.51 ± 6.48 beats per minute (bpm) in Group A and 80.11 ± 5.62 bpm in Group B (p = 0.3377). Throughout the intraoperative period, HR remained stable and similar between groups, ranging from 74 to 77 bpm. The lowest HR was recorded at five minutes (74.71 ± 7.07 bpm in Group A vs. 73.31 ± 6.18 bpm in Group B, p = 0.3809), and the highest at seven minutes (77.34 ± 6.87 bpm vs. 76.06 ± 5.23 bpm, p = 0.3838). The p-value is based on Student’s t-test (Table 5 & Figure 3).

**Table 5:** Intraoperative HR (bpm) The p-value is based on Student’s t-test. HR: heart rate; min: minutes; bpm: beats per minute

HR	Group A		Group B		p-value
	Mean	SD (+/ -)	Mean	SD (+/ -)	
Baseline	81.51	6.48	80.11	5.62	0.34
5 min	74.71	7.07	73.31	6.18	0.38
7 min	77.34	6.87	76.06	5.23	0.38
9 min	77.51	6.24	76.03	5.71	0.30
11 min	77.89	6.61	76.43	5.66	0.32
13 min	77.71	6.19	76.11	5.34	0.25
15 min	76.23	7.72	74.54	6.51	0.32
25 min	75.09	7.91	74.66	6.69	0.81
35 min	75.97	6.93	74.71	6.48	0.43
45 min	75.83	6.92	74.40	6.15	0.36
60 min	75.46	7.34	74.34	7.02	0.52
75 min	75.26	7.43	74.66	5.98	0.71
90 min	76.00	7.34	74.09	6.54	0.25
115 min	76.34	6.96	74.83	6.67	0.36
140 min	76.17	7.44	74.86	6.61	0.44
165 min	75.83	7.63	74.00	6.61	0.29
190 min	75.75	8.02	74.21	6.31	0.37

SpO_2_ remained consistently high throughout the intraoperative period, with values fluctuating between 99.3%-99.6% in both groups. At five minutes, a statistically significant difference was observed (99.29 ± 0.46% in Group A vs. 99.63 ± 0.49% in Group B, p = 0.0039). The p-value is based on Student’s t-test (Table 6 & Figure 3).

**Table 6:** Intraoperative SpO2 (%) The p-value is based on Student’s t-test. min: minutes

SpO_2_	Group A		Group B		p-value
	Mean	SD (+/-)	Mean	SD (+/-)	
Baseline	99.49	0.51	99.40	0.50	0.46
5 min	99.57	0.50	99.43	0.50	0.25
7 min	99.49	0.51	99.51	0.51	0.87
9 min	99.57	0.50	99.54	0.51	0.80
11 min	99.54	0.51	99.54	0.51	1
13 min	99.46	0.51	99.51	0.51	0.68
15 min	99.54	0.51	99.51	0.51	0.80
25 min	99.43	0.50	99.46	0.51	0.80
35 min	99.57	0.50	99.60	0.50	0.80
45 min	99.54	0.51	99.40	0.50	0.25
60 min	99.57	0.50	99.57	0.50	1
75 min	99.54	0.51	99.63	0.49	0.45
90 min	99.57	0.50	99.40	0.50	0.16
115 min	99.54	0.51	99.51	0.51	0.80
140 min	99.57	0.50	99.49	0.51	0.51
165 min	99.57	0.50	99.43	0.50	0.25
190 min	99.41	0.50	99.50	0.51	0.46

Baseline RR was 15.31 ± 0.76 breaths per minute in Group A and 15.20 ± 0.72 breaths per minute in Group B (p = 0.5363). RR values fluctuated slightly intraoperatively, with no significant differences at any time points. The p-value is based on Student’s t-test (Table 7 & Figure 3)

**Table 7:** Intraoperative RR (breaths per minute) The p-value is based on Student’s t-test. RR: respiratory rate; min: minutes

RR	Group A		Group B		p-value
	Mean	SD (+/-)	Mean	SD (+/-)	
Baseline	15.31	0.76	15.20	0.72	0.54
5 min	14.86	0.91	15.23	0.73	0.06
7 min	15.06	0.80	14.97	0.79	0.64
9 min	14.91	0.89	14.89	0.76	0.92
11 min	14.91	0.74	14.94	0.84	0.87
13 min	14.69	0.80	15.03	0.75	0.07
15 min	14.94	0.87	15.03	0.82	0.65
25 min	14.94	0.87	15.00	0.80	0.76
35 min	15.11	0.68	15.23	0.73	0.48
45 min	14.91	0.74	14.94	0.84	0.87
60 min	14.94	0.84	15.17	0.71	0.22
75 min	14.94	0.73	14.91	0.78	0.87
90 min	14.97	0.86	15.09	0.82	0.55
115 min	15.06	0.91	14.83	0.79	0.27
140 min	14.97	0.86	15.03	0.79	0.76
165 min	14.77	0.81	14.94	0.84	0.39
190 min	15.06	0.80	15.00	0.85	0.76

**Figure 4:**
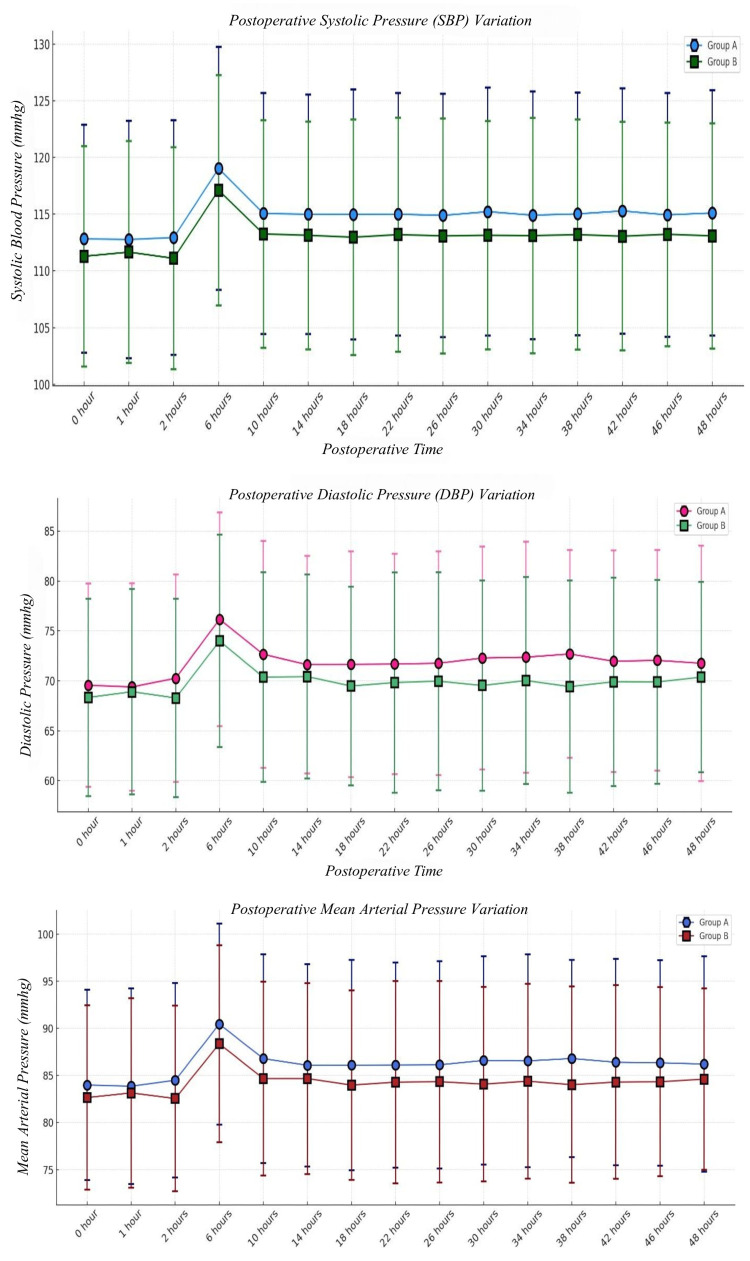
Postoperative systolic, diastolic, and mean arterial blood pressures The p-value is based on Student’s t-test. min: minutes

At 0 hours postoperatively, the mean SBP was 112.83 ± 10.06 mmHg in Group A and 111.29 ± 9.70 mmHg in Group B (p = 0.5166), indicating no statistically significant difference at baseline. Over the next 48 hours, SBP values remained within a stable range of 112-115 mmHg in both groups. The highest recorded SBP was at six hours postoperatively, with Group A at 119.03 ± 10.72 mmHg and Group B at 117.11 ± 10.15 mmHg (p = 0.4443). By 48 hours, SBP had slightly decreased to 115.11 ± 10.81 mmHg in Group A and 113.09 ± 9.92 mmHg in Group B (p = 0.4683). The p-value is based on Student’s t-test. Since all p-values remained above 0.05, no significant differences were observed between the groups, indicating that both maintained stable postoperative systolic blood pressure without significant fluctuations or hemodynamic instability (Table 8 & Figure 4).

**Table 8:** Postoperative SBP (mmHg) The p-value is based on Student’s t-test. SBP: systolic blood pressure; min: minutes

SBP	Group A		Group B		p-value
	Mean	SD (+/-)	Mean	SD (+/-)	
0 hour	112.83	10.06	111.29	9.70	0.52
1 hour	112.77	10.46	111.66	9.78	0.65
2 hours	112.94	10.32	111.11	9.79	0.45
6 hours	119.03	10.72	117.11	10.15	0.44
10 hours	115.06	10.61	113.26	10.03	0.47
14 hours	115.00	10.56	113.14	10.04	0.45
18 hours	114.97	11.01	112.97	10.38	0.43
22 hours	115.00	10.69	113.20	10.33	0.47
26 hours	114.89	10.72	113.09	10.37	0.48
30 hours	115.23	10.94	113.14	10.06	0.41
34 hours	114.91	10.91	113.11	10.37	0.48
38 hours	115.03	10.70	113.20	10.13	0.46
42 hours	115.29	10.82	113.06	10.06	0.37
46 hours	114.94	10.76	113.23	9.85	0.49
48 hours	115.11	10.81	113.09	9.92	0.42

The baseline postoperative DBP at 0 hours was 69.57 ± 10.17 mmHg in Group A and 68.34 ± 9.88 mmHg in Group B (p = 0.6095), showing no initial difference between the groups. Throughout the 48 hours, DBP values ranged between 69 and 76 mmHg, with the highest value recorded at six hours (76.17 ± 10.70 mmHg in Group A vs. 74.00 ± 10.65 mmHg in Group B, p = 0.3981). At 48 hours, DBP was 71.77 ± 11.79 mmHg in Group A and 70.37 ± 9.54 mmHg in Group B (p = 0.6930), showing no statistically significant difference. The p-value is based on Student’s t-test (Table 9 & Figure 4).

**Table 9:** Postoperative DBP (mmHg) The p-value is based on Student’s t-test. DBP: diastolic blood pressure; min: minutes

DBP	Group A		Group B		p-value
	Mean	SD (+/-)	Mean	SD (+/-)	
0 hour	69.57	10.17	68.34	9.88	0.61
1 hour	69.40	10.38	68.91	10.27	0.84
2 hours	70.26	10.38	68.29	9.95	0.42
6 hours	76.17	10.70	74.00	10.65	0.39
10 hours	72.66	11.35	70.37	10.51	0.38
14 hours	71.63	10.90	70.43	10.21	0.64
18 hours	71.66	11.30	69.49	9.96	0.39
22 hours	71.69	11.03	69.83	11.01	0.48
26 hours	71.77	11.20	69.97	10.91	0.49
30 hours	72.29	11.16	69.54	10.53	0.29
34 hours	72.37	11.55	70.03	10.37	0.37
38 hours	72.69	10.40	69.43	10.64	0.19
42 hours	71.97	11.09	69.91	10.44	0.43
46 hours	72.06	11.03	69.89	10.20	0.39
48 hours	71.77	11.79	70.37	9.54	0.59

The postoperative MAP was also well-maintained and comparable between the groups. At 0 hours, Group A had a MAP of 83.99 ± 10.10 mmHg, while Group B had 82.66 ± 9.78 mmHg (p = 0.5775), indicating similar initial perfusion pressures. The highest MAP was at six hours, where Group A had 90.46 ± 10.67 mmHg and Group B had 88.37 ± 10.44 mmHg (p = 0.4104). By 48 hours, MAP had stabilized at 86.22 ± 11.43 mmHg in Group A and 84.61 ± 9.63 mmHg in Group B (p = 0.6023). The p-value is based on Student’s t-test.

These findings indicate that postoperative mean blood pressures remained consistent across both groups, with no significant hemodynamic derangements observed (Table 10 & Figure 4).

**Table 10:** Postoperative MAP (mmHg) The p-value is based on Student’s t-test. MAP: mean arterial pressure; min: minutes

MAP	Group A		Group B		p-value
	Mean	SD (+/-)	Mean	SD (+/-)	
0 hour	83.99	10.10	82.66	9.78	0.58
1 hour	83.86	10.38	83.16	10.06	0.77
2 hours	84.49	10.32	82.56	9.86	0.43
6 hours	90.46	10.67	88.37	10.44	0.41
10 hours	86.79	11.07	84.67	10.31	0.41
14 hours	86.09	10.75	84.67	10.12	0.57
18 hours	86.10	11.16	83.98	10.06	0.41
22 hours	86.12	10.88	84.29	10.75	0.48
26 hours	86.14	11.01	84.34	10.69	0.49
30 hours	86.60	11.05	84.08	10.34	0.33
34 hours	86.55	11.30	84.39	10.33	0.41
38 hours	86.80	10.46	84.02	10.42	0.27
42 hours	86.41	10.95	84.30	10.27	0.41
46 hours	86.35	10.90	84.33	10.04	0.42
48 hours	86.22	11.43	84.61	9.63	0.53

**Figure 5:**
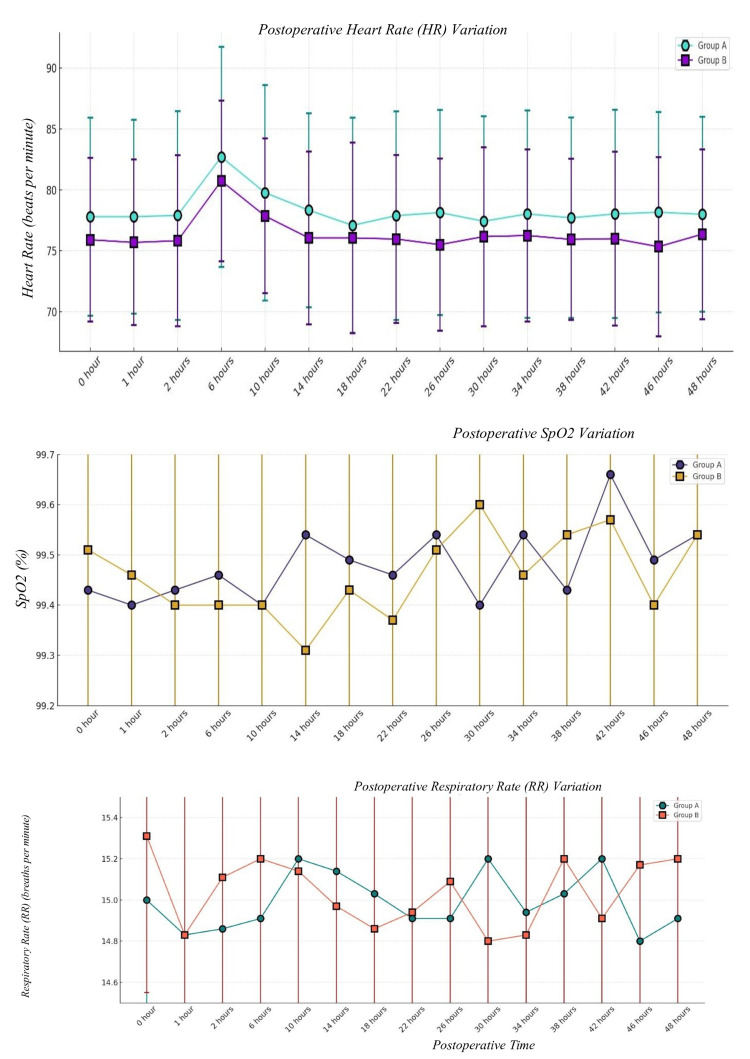
Postoperative heart rate, Spo2, and respiratory rate The p-value is based on Student’s t-test. min: minutes

The HR trend postoperatively followed a stable trajectory in both groups. At 0 hours, the HR was 77.80 ± 8.12 bpm in Group A and 75.91 ± 6.73 bpm in Group B (p = 0.2929), showing no significant difference at baseline. The highest HR was observed at six hours, with 82.71 ± 9.03 bpm in Group A vs. 80.74 ± 6.59 bpm in Group B (p = 0.3012), though this variation was not statistically significant. By 48 hours, HR remained stable at 78.00 ± 8.00 bpm in Group A and 76.37 ± 6.97 bpm in Group B (p = 0.4267). The p-value is based on Student’s t-test. The HR stability suggests that both groups had similar autonomic responses postoperatively, without significant tachycardia or bradycardia (Table 11 & Figure 5).

**Table 11:** Postoperative HR (bpm) The p-value is based on Student’s t-test. HR: heart rate; min: minutes; bpm: beats per minute

HR	Group A		Group B		p-value
	Mean	SD (+/ -)	Mean	SD (+/ -)	
0 hour	77.80	8.12	75.91	6.73	0.29
1 hour	77.80	7.94	75.69	6.80	0.24
2 hours	77.91	8.57	75.83	7.01	0.27
6 hours	82.71	9.03	80.74	6.59	0.30
10 hours	79.77	8.84	77.86	6.35	0.30
14 hours	78.34	7.97	76.06	7.11	0.21
18 hours	77.09	8.83	76.06	7.81	0.61
22 hours	77.89	8.55	75.97	6.89	0.30
26 hours	78.14	8.41	75.51	7.06	0.16
30 hours	77.43	8.61	76.17	7.34	0.51
34 hours	78.03	8.51	76.26	7.07	0.35
38 hours	77.71	8.24	75.94	6.61	0.32
42 hours	78.03	8.54	76.00	7.12	0.28
46 hours	78.17	8.22	75.34	7.37	0.13
48 hours	78.00	8.00	76.37	6.97	0.37

Postoperative SpO_2_ at various time periods is comparable but statistically insignificant. The p-value is based on Student’s t-test (Table 12 & Figure 5).

**Table 12:** Postoperative SpO2 (%) The p-value is based on Student’s t-test. min: minutes

SpO_2_	Group A		Group B		p-value
	Mean	SD (+/-)	Mean	SD (+/-)	
0 hour	99.43	0.50	99.51	0.51	0.51
1 hour	99.40	0.50	99.46	0.51	0.62
2 hours	99.43	0.50	99.40	0.50	0.80
6 hours	99.46	0.51	99.40	0.50	0.62
10 hours	99.40	0.50	99.40	0.50	1
14 hours	99.54	0.51	99.31	0.47	0.06
18 hours	99.49	0.51	99.43	0.50	0.62
22 hours	99.46	0.51	99.37	0.49	0.45
26 hours	99.54	0.51	99.51	0.51	0.80
30 hours	99.40	0.50	99.60	0.50	0.09
34 hours	99.54	0.51	99.46	0.51	0.51
38 hours	99.43	0.50	99.54	0.51	0.36
42 hours	99.66	0.48	99.57	0.50	0.44
46 hours	99.49	0.51	99.40	0.50	0.46
48 hours	99.54	0.51	99.54	0.51	1

Postoperative RR at various time periods is comparable but statistically insignificant. The p-value is based on Student’s t-test. The p-value is based on Student’s t-test (Table 13 & Figure 5).

**Table 13:** Postoperative RR (breaths per minute) The p-value is based on Student’s t-test. RR: respiratory rate; min: minutes

RR	Group A		Group B		p-value
	Mean	SD (+/-)	Mean	SD (+/-)	
0 hour	15.00	0.80	15.31	0.76	0.10
1 hour	14.83	0.82	14.83	0.79	1
2 hours	14.86	0.73	15.11	0.76	0.16
6 hours	14.91	0.82	15.20	0.87	0.16
10 hours	15.20	0.83	15.14	0.85	0.76
14 hours	15.14	0.81	14.97	0.71	0.35
18 hours	15.03	0.86	14.86	0.85	0.41
22 hours	14.91	0.85	14.94	0.91	0.88
26 hours	14.91	0.82	15.09	0.82	0.36
30 hours	15.20	0.80	14.80	0.90	0.06
34 hours	14.94	0.84	14.83	0.82	0.58
38 hours	15.03	0.79	15.20	0.80	0.37
42 hours	15.20	0.72	14.91	0.85	0.13
46 hours	14.80	0.87	15.17	0.75	0.06
48 hours	14.91	0.85	15.20	0.72	0.13

**Figure 6:**
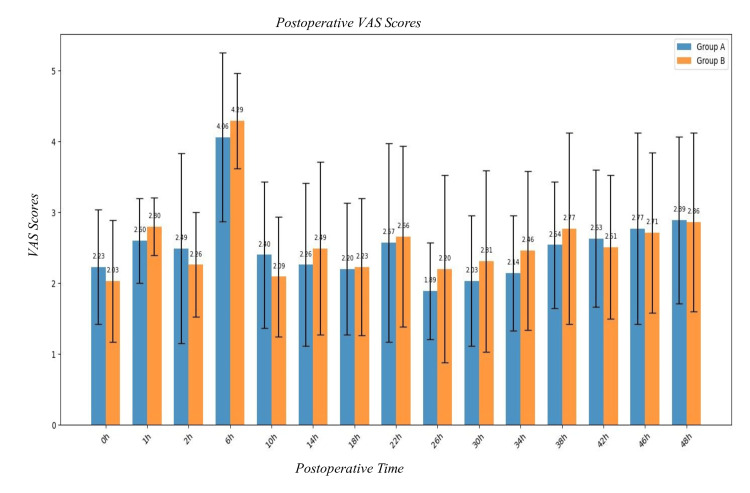
Postoperative Visual Analog Scores (VAS) score The p-value is based on Student’s t-test.

Postoperative VAS scores comparing Group A and Group B at multiple time intervals over 48 hours. Both groups show a peak in pain scores at six hours, followed by a gradual decrease. Postoperative VAS trajectories were comparable from 0 to 48 hours; Group A was lower at 9/15 time points and higher at 6/15, with no statistically significant differences (all p>0.05; e.g., six hours: 4.06±1.19 vs 4.29±0.67, p=0.32), indicating comparable postoperative analgesic efficacy between the groups. The p-value is based on Student’s t-test (Table 14 & Figure 6).

**Table 14:** Postoperative Visual Analog Scores (VAS) scores The p-value is based on Student’s t-test.

VAS	Group A		Group B		p-value
	Mean	SD (+/-)	Mean	SD (+/-)	
0 hour	2.23	0.81	2.03	0.86	0.32
1 hour	2.60	0.60	2.80	0.41	0.11
2 hours	2.49	1.34	2.26	0.74	0.37
6 hours	4.06	1.19	4.29	0.67	0.32
10 hours	2.40	1.03	2.09	0.85	0.17
14 hours	2.26	1.15	2.49	1.22	0.42
18 hours	2.20	0.93	2.23	0.97	0.89
22 hours	2.57	1.40	2.66	1.28	0.78
26 hours	1.89	0.68	2.20	1.32	0.22
30 hours	2.03	0.92	2.31	1.28	0.29
34 hours	2.14	0.81	2.46	1.12	0.17
38 hours	2.54	0.89	2.77	1.35	0.40
42 hours	2.63	0.97	2.51	1.01	0.61
46 hours	2.77	1.35	2.71	1.13	0.84
48 hours	2.89	1.18	2.86	1.26	0.92

**Figure 7:**
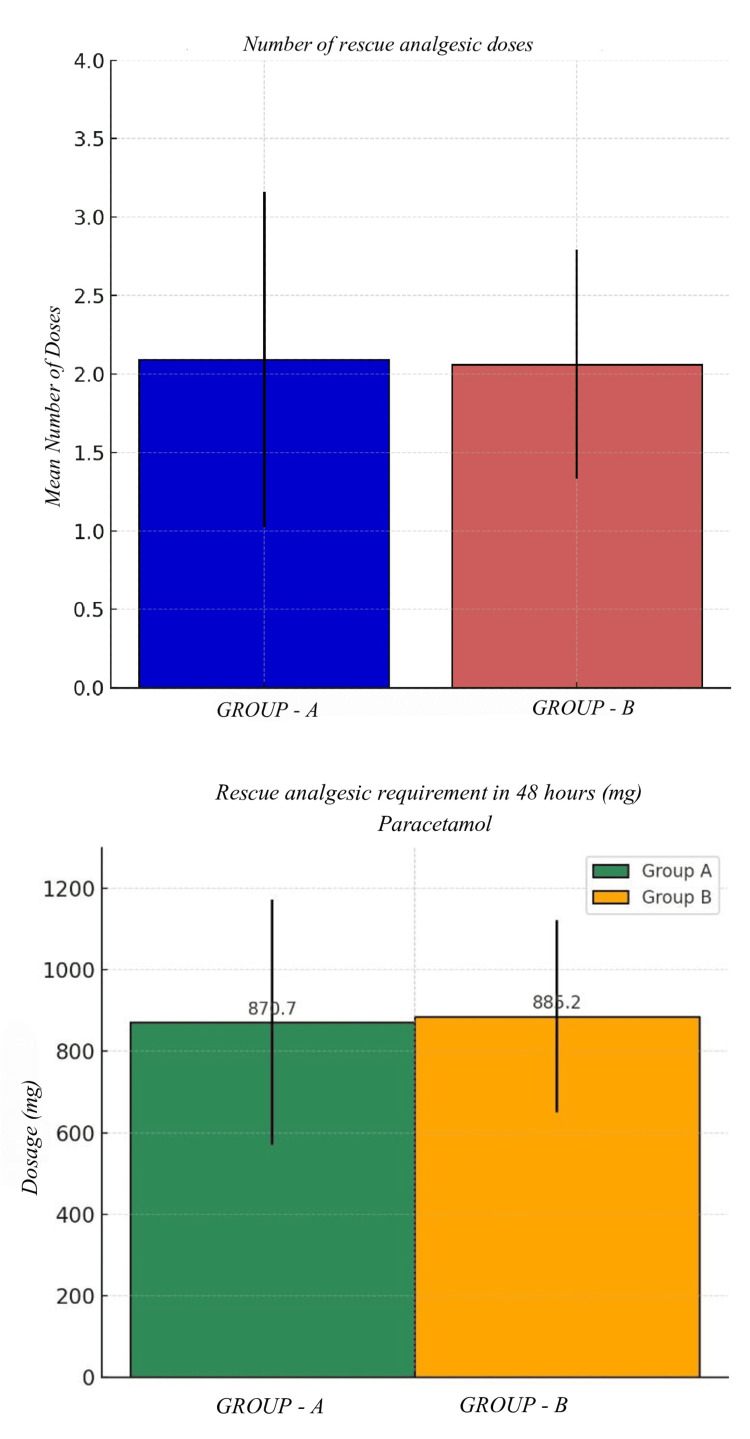
Number of rescue analgesia doses and requirements The p-value is based on Student’s t-test.

The mean number of rescue analgesia doses was similar in both groups (Group A: 2.09 ± 1.07, Group B: 2.06 ± 0.73), with no statistically significant difference observed (p = 0.90). This suggests that both groups had comparable postoperative analgesic requirements. The p-value is based on Student’s t-test (Table 15 & Figure 7).

**Table 15:** Number of rescue analgesic doses The p-value is based on Student’s t-test.

Doses	Group A		Group B		p-value
	Mean	SD (+/ -)	Mean	SD (+/ -)	
	2.09	1.07	2.06	0.73	0.89

The mean consumption of paracetamol was comparable between Group A (870.68 ± 301.11 mg) and Group B (885.21 ± 235.47 mg), with no statistically significant difference (p = 0.82). The p-value is based on Student’s t-test (Table 16 & Figure 7).

**Table 16:** Rescue analgesic requirement in 48 hours (mg) The p-value is based on Student’s t-test.

Rescue Dosage (mg)	Group A		Group B		p -value
	Mean	SD (+/ -)	Mean	SD (+/ -)	
Paracetamol	870.68	301.11	885.21	235.47	

## Discussion

Postoperative pain management is essential for recovery and rehabilitation following knee surgeries, which often result in significant postoperative pain due to extensive manipulation of soft tissues and bones. Inadequate analgesia can hinder early mobilization, prolong rehabilitation, and extend hospital stay. CFNB and CEA are used among the various regional anesthesia techniques employed for postoperative pain control.

The primary objective of our study is to compare postoperative VAS scores at predetermined time intervals between CFNB and CEA following knee surgeries. Both groups exhibited peak pain (VAS > 4) scores at the sixth hour, followed by a gradual decrease. Postoperative VAS trajectories were comparable from 0 to 48 hours; Group A was lower at 9/15 time points and higher at 6/15, with no statistically significant differences (all p>0.05; e.g., six hours: 4.06±1.19 vs 4.29±0.67, p=0.32). indicating comparable postoperative analgesic efficacy.

The secondary objective was to compare the total number of rescue analgesics required between the two groups. The mean number of doses of rescue analgesia was similar in both groups (Group A: 2.09 ± 1.07, Group B: 2.06 ± 0.73), with no statistically significant difference observed (p = 0.90). This suggests that both groups had comparable postoperative analgesic requirements.

Our study was consistent with findings from a clinical trial by Vishwanatha et al. [12], which compared continuous femoral nerve blockade with continuous epidural analgesia for postoperative pain relief in knee surgeries. Postoperatively, continuous infusion of 0.0625% bupivacaine and 2 μg/ml fentanyl was started at 5 ml/h for 72 hours in both groups, and the study concluded that both CFNB and CEA provided effective analgesia with comparable VAS scores at various time intervals, which were not statistically significant (p value > 0.05), and there was no significant difference between the groups regarding the required doses of rescue analgesics.

Harshil et al. [13] analyzed a study to compare CFNB and continuous epidural infusion (CEI) with 0.2% ropivacaine for postoperative analgesia and knee rehabilitation after total knee arthroplasty. An infusion was started at 6 ml/h postoperatively when VAS was ≥4. The dose was titrated to keep the VAS <4. If VAS >4 despite the maximum rate of infusion, an injection of diclofenac sodium 1.5 mg/kg was given intravenously as a rescue analgesic. The study concluded that there was no statistically significant difference in mean VAS scores (p value > 0.05), and the requirement for rescue analgesics was similar between the two groups.

A similar study by Shanthini et al. [14] compared CFNB and CEA for postoperative pain management in patients undergoing elective total knee replacement surgery. This study concluded that both CFNB (p-value = 0.5) and CEA (p-value = 0.694) provided equally effective postoperative pain relief, as measured by VAS, and there was no significant difference in the need for supplemental analgesics in both groups.

Correspondingly, in a noteworthy study, Singelyn et al. [15] compared the effects of intravenous patient-controlled analgesia (PCA) with morphine, CEA, and continuous three-in-one block on postoperative pain and knee rehabilitation after unilateral TKA. Postoperative analgesia was provided with IV PCA with morphine in Group A, continuous three-in-one block in Group B, and epidural analgesia in Group C. The study concluded that loco-regional analgesia techniques (continuous three-in-one block or epidural analgesia) are superior to IV PCA with morphine for pain relief and early postoperative knee rehabilitation.

In another study conducted by Barrington et al. [16], they compared continuous femoral nerve blockade and continuous epidural analgesia for postoperative pain management after total knee replacement surgery. The study concluded that there were no significant differences in VAS scores between the groups, and CFNB was a feasible alternative to CEA, offering comparable pain relief.

Sundarathiti et al. [17] compared CFNB and CEI for postoperative analgesia and knee rehabilitation after TKA. The CEI group received a continuous infusion of 0.125% levobupivacaine with morphine 0.0125 mg/ml (4 ml/h), while the CFNB group received 0.125% levobupivacaine (8 ml/h) for 48 hours. When VAS >4, despite infusion, an injection of tramadol 50mg IV was given as a rescue analgesic. The study concluded that there were no significant differences in the VAS scores for the first hour and at 12-72 hours between the two groups. At six to 12 hours, the VAS scores were significantly greater in the CFNB group compared with the CEI group (at six hours, p-value = 0.001 & at 12 hours, p-value = 0.004). Rescue analgesic requirement was more in the CFNB group (median = 150 mg, range 0-350 mg) compared with the CEI group (median = 50 mg, range 0-150 mg, p = 0.001). The study concluded that CFNB is the optimal technique for postoperative analgesia in TKA, offering fewer side effects and greater patient satisfaction. CEI provided superior pain relief during the six- to 12-hour postoperative window but is associated with more side effects like hypotension, motor block, urinary retention, pruritus, infection, and catheter migration or dislodgement.

A clinical trial by Shanthanna et al. [18] compared CFNB and CEA for postoperative pain relief following total knee replacement. Infusion of a mixture of 0.125% bupivacaine with 2 mcg/ml of fentanyl was started in both groups postoperatively. The initial rate of infusion was set at 8 mL/h in both groups, which was then titrated according to the VAS score. Which were significantly high (P=0.001) in the femoral group at six hours, after which there was a declining trend, and scores were essentially similar from 24 hours (P=0.825). The use of rescue analgesia was also higher in the femoral group, but the difference was not statistically significant (p value > 0.05). After the first six hours, the usage of rescue analgesics was nearly the same in both groups.

The clinical implications of these findings from the above-mentioned studies suggest that both CFNB and CEA are safe and effective, and the choice should be guided by patient-specific considerations, including contraindications, risk of hypotension, and the need for motor function preservation.

## Conclusions

This study demonstrated that both CFNB and CEA provide effective postoperative analgesia in knee surgeries, with comparable hemodynamic stability and minimal adverse effects. The analysis of demographic and intraoperative parameters, including blood pressure, heart rate, and analgesic requirements, revealed no statistically significant differences between the two groups. While CFNB offers the advantage of preserving motor function and reducing the risk of hypotension, CEA remains a reliable option with broader analgesic coverage.

Intraoperative hemodynamic parameters, including SBP, DBP, and MAP, remained stable and comparable across both groups at various time points. The statistical analysis revealed no significant variations, indicating that both groups maintained similar intraoperative hemodynamic stability. Additionally, there was no significant difference in the duration of surgery between the groups. The choice of analgesic technique should be tailored to individual patient needs, considering clinical scenarios and potential benefits.

Overall, the findings suggest that the interventions or treatments compared in this study did not result in any significant differences in intraoperative hemodynamics or surgical duration. This reinforces the safety and efficacy of both approaches under investigation, providing valuable insights for clinical decision-making. Further studies with larger sample sizes may help to validate these findings and explore additional parameters influencing patient outcomes.
